# Social Questions and the Medical Profession

**Published:** 1908-01-25

**Authors:** 


### The Hospital

A Journal of

The Medical Sciences and Hospital Administration.

New Series. No. 45, Vol. II. [No. 1117, Vol. XLIII.] SATURDAY, JANUARY 25, 1908.

### Some Social Questions and the Medical Profession.

The contact of medicine with important social problems is becoming an increasingly intimate one. It has long been recognised that the promotion of the health of the community by the methods of municipal hygiene and by the isolation of cases of infectious disease falls well within the area of public activity, and charges incurred for this purpose are nowadays met freely and without discussion from public funds. Signs are not wanting that the near future will bring proposals to apply this principle over a much wider field than it at present occupies. In these proposals the interests of medical practitioners are closely concerned, and it will be well for the profession to consider in advance how the claims of the community and the rights of individuals are to be reconciled at any point where they may come into conflict. Unless some just and adequate policy is prepared, there will, we fear, be both confusion and uncertainty at the very moment when decision in word and act is essential. The experience of the past teaches only too plainly that in various public and corporate movements the legitimate rights of the profession are apt to receive but scant consideration. From the same source, too, come warnings of the helplessness of medical practitioners in consequence of their want of union and organisation. The future, it cannot be doubted, will repeat this experience, unless the conditions which at present exist in the profession are essentially changed. Such a change can only be brought about by a recognition of the tendencies of the times, and by a determination on the part of the great body of the profession to speak with a united voice on questions in which the interests of practitioners are directly concerned. This by no means implies that some narrow and selfish policy is to be devised and followed. On the contrary, we believe that no body of men is more ready than the medical profession to place the claims of public welfare in their legitimate place. But there are rights which pertain to every individual who renders service to the common good, and it is often necessary to affirm these in order that justice may be done. Such affirmation to be effective must be supported by adequate reasons and by skilful organisation. The cause must, of course, be a just one, but to hope for its success without forcible argument and apt presentation is a fond and foolish delusion.

There may now be mentioned one or two questions which the immediate future will certainly force on the attention of the medical profession. First among these is the position created by the compulsory examination of children in attendance at elementary schools. This duty will mean both responsible work and the expenditure of time, and such claims demand appropriate remuneration. Already the suggestion has been advanced that these duties may be committed to women practitioners, and that to such practitioners a smaller salary may be paid than would be necessary in the case of men. The leaders, as they may be called, of the medical women have spoken strongly in opposition to this suggestion, and it is to be hoped that the position thus defined will be firmly maintained. Another outcome of the medical inspection of school children will be the delicate question of the treatment of those whose health renders the successful prosecution of their studies impossible. This will particularly arise in connection with defects or diseases of the special-sense organs and of the teeth. Unless care is taken we have little doubt that large numbers of such children will be introduced to the out-patient departments of the hospitals, and that in this way the hospital habit will be even more widely extended than it is at present. Whether treatment in these cases should or should not be provided at the expense of the public is a political question, with which in these columns we have no concern. But we are quite sure it ought not to be provided at the expense of one section of the community—namely, the medical profession. Doctors, like other citizens, must meet their share of public charges, but they ought not to be asked to shoulder the entire burden.

Another public question in which the profession is concerned is the suggested provision of a public fund for the training of midwives. We are not now arguing either for or against this proposal. But it touches the interest of many practitioners, and it is one on which the profession ought to have a policy that, at the appropriate time, may be duly presented and argued. Again, from one of the northern cities comes a proposal to establish, at the expense of the municipality, maternity homes, where women may be received and attended during confinement. It is urged in support of this that the conditions existing among the poorer classes make it difficult or impossible to ensure asepsis during labour, more especially if instrumental interference is needed, and that to meet this position homes for the reception of poor women should be established at the public expense. Here again is a proposal which has a socio-political aspect, on which opinions may differ. But the proposal concerns directly those engaged in medical practice, and it is a necessity that it shall be discussed from their point of view.

The claim for professional union is often accompanied by the suggestion that the absence of it is due to the apathy of the general practitioner. With all deference we will suggest that some responsibility must lie with the more prominent members of the profession. In two recent instances Acts of Parliament have placed duties on medical practitioners without any provision for the payment of an appropriate fee. How many of our recognised authorities have displayed the slightest interest in the positions thus created? In this article we have indicated some few questions the settlement of which is now close at hand. We suggest that to this settlement on lines just to the profession it is the duty of every member of the profession to contribute, and this, whether his personal interests are or are not directly concerned.

